# Clitoral leiomyoma in a premenopausal woman: a case report

**DOI:** 10.1186/s12905-020-00959-x

**Published:** 2020-05-01

**Authors:** Gianmarco Taraschi, Diego Aguiar, Jean Christophe Tille, Patrick Petignat, Jasmine Abdulcadir

**Affiliations:** 1 Department of Obstetrics and Gynaecology, Geneva University Hospitals, 30 Bld de la Cluse, 1211 Geneva, Switzerland; 2 Division of Clinical Pathology, Geneva University Hospital, 1 rue Michel-Servet, 1205 Geneva, Switzerland

**Keywords:** Extrauterine leiomyoma, Alport’s syndrome, Vulvar lesions

## Abstract

**Background:**

Extrauterine leiomyomata is an uncommon lesion that can lead to several problems of differential diagnosis, especially when localized in the external genitalia. There are few reports in the English literature and a novel association with Alport’s syndrome has been investigated since the 1980s.

**Case presentation:**

Here, we describe the case of a premenopausal woman who presented with an indolent swelling of the right interlabial fossa that resulted in a Bartholin cyst. In addition to this cyst, a benign leiomyoma of the right side of the clitoris was also found and removed. Our patient refused any further examination, although she was informed that genetic counselling could be organized to rule out an association with Alport’s syndrome.

**Conclusions:**

Extrauterine leiomyomata localized in the external genitalia is an uncommon lesion arising from smooth muscle cells around vascular epithelium or erectile tissue. Since an association between extrauterine leiomyomata and Alport’s syndrome has been described, genetic testing can be proposed to these patients. Upper intestinal tract symptoms such as dysphagia should prompt a gastroenterological evaluation as an association with an esophageal leiomyomatosis has been described.

## Background

Extrauterine leiomyomata localized in the external genitalia is an uncommon lesion arising from smooth muscle cells around vascular epithelium or erectile tissue [1]. The most common extrauterine sites of the disease are the upper digestive tract or the lower respiratory tract, but if the female genital tract is involved, the most frequent localization is the clitoris [2]. While therapeutic management is clear, with surgical excision as the treatment of choice, little is known about the appropriate long-term management, particularly regarding association with other diseases like Alport’s Syndrome. Here, we present the case of a 39-year-old woman with a chronic indolent swelling of the right labial fossa who was referred for cystectomy.

## Case presentation

**Fig. 1:**
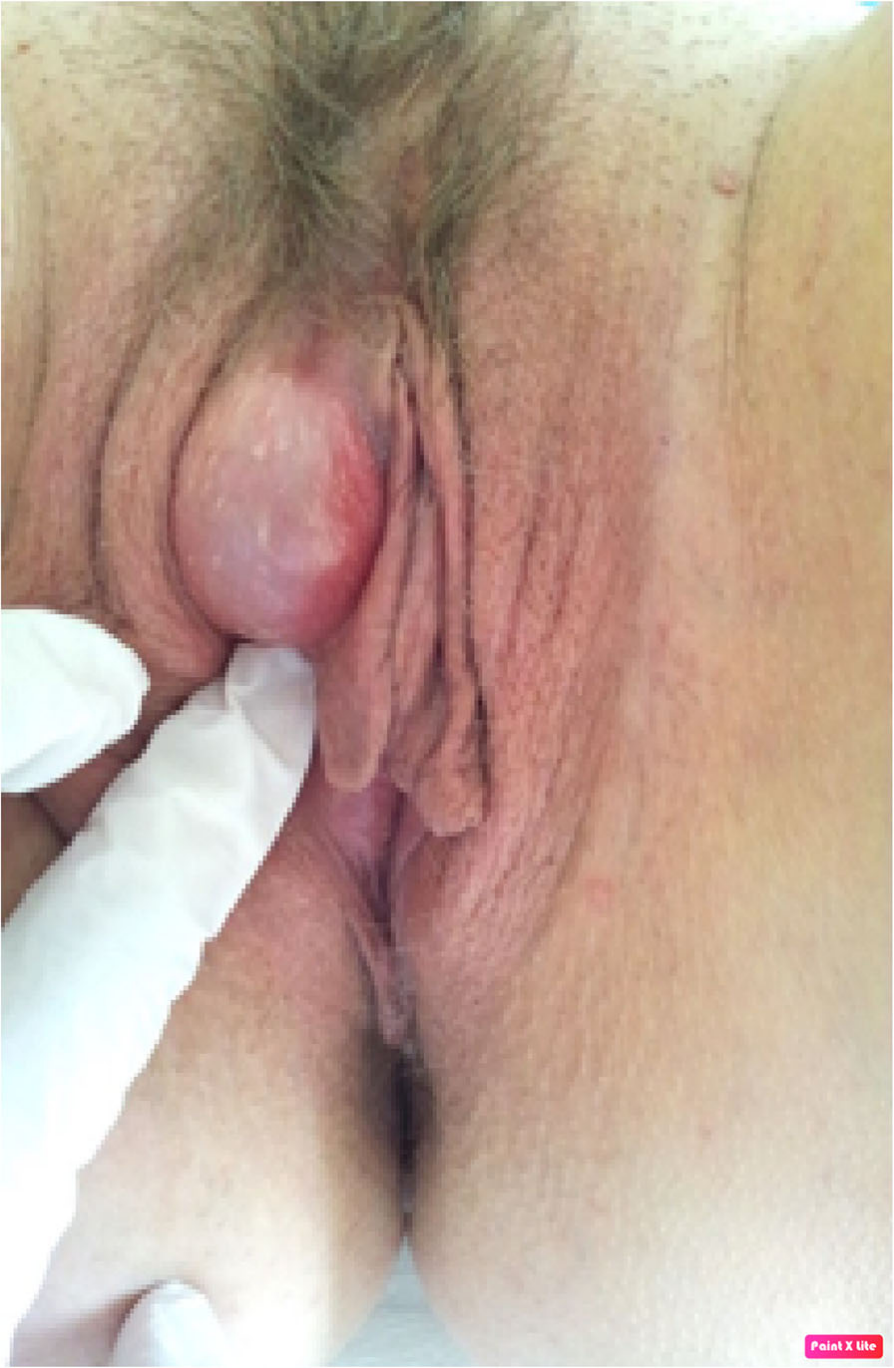
Physical examination, soft bi-lobated fluid-filled swelling of the right interlabial fossa slightly diverting the clitoris on the opposite side. The pathologic examination showed a Bartholin’s gland cyst, which was surprising considering the anatomical localization of this lesion

A 39-year-old, Caucasian 1G0P0A0T woman at 5 Gestational Weeks (GW) consulted with a chief complaint of a non-desired pregnancy. She also complained about an indolent swelling of the right interlabial fossa measuring 7 × 3 × 3 cm. The mass has not significantly changed in 9 years. Her medical history was unremarkable. Her sexual history revealed no dyspareunia and physiological sexual response. On physical examination, we observed a soft, painless bi-lobated fluid-filled swelling of the right interlabial fossa slightly diverting the clitoris on the opposite side (Fig. 1). Speculum, digital and general examination were normal.

**Fig. 2:**
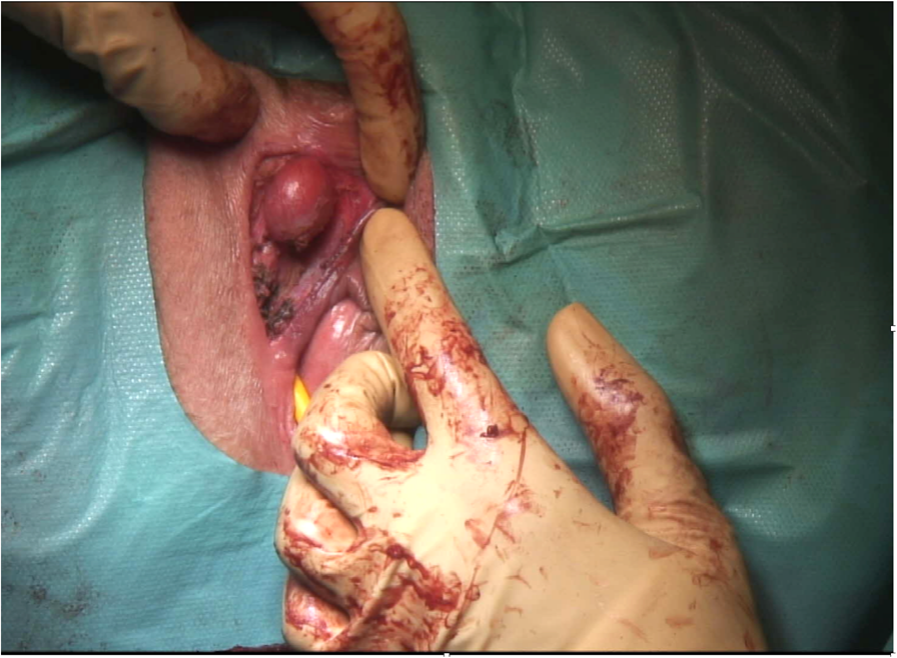
Macroscopic view of the clitoridal mass. (1 square is 1 cm)

**Fig. 3:**
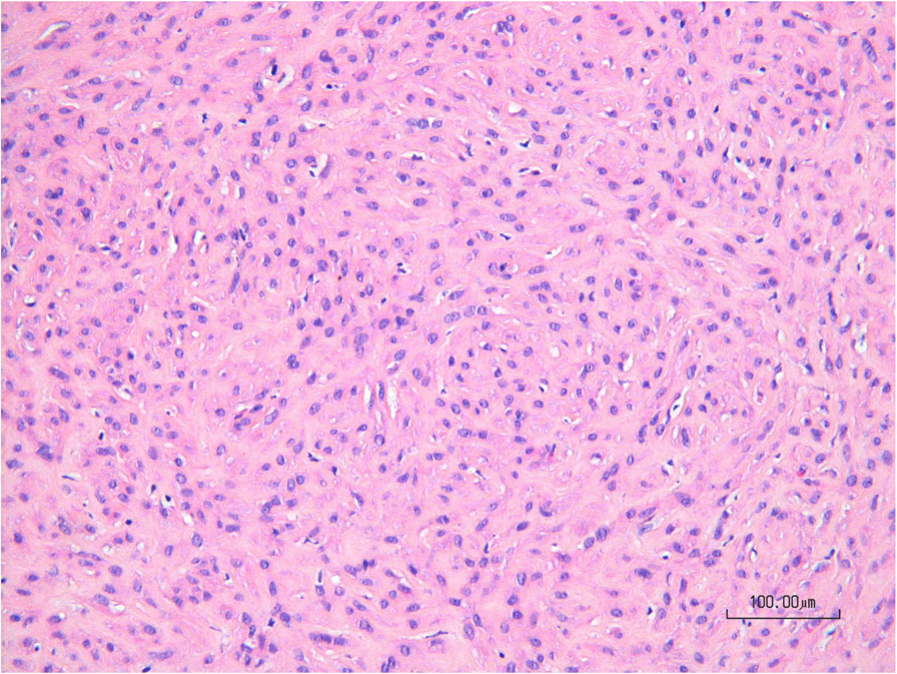
Intra-operative image of the leiomyoma arising from the right side of clitoris after excision of the first Bartholin’s gland cyst

**Fig. 4:**
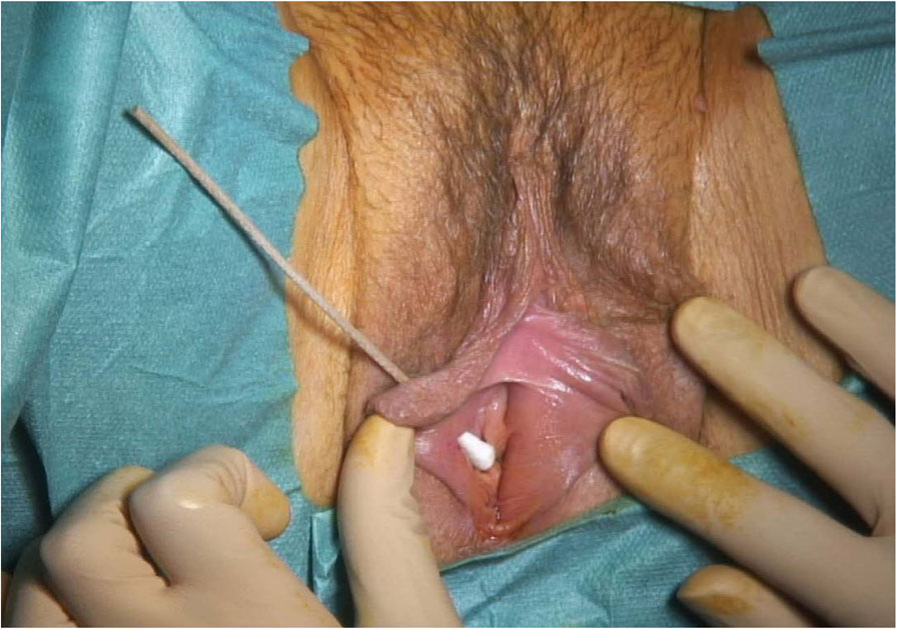
Microscopic aspect of the clitoridal mass showing intersecting fascicles of spindled cells intermixed with collagen (HE, 200x)

**Fig. 5:**
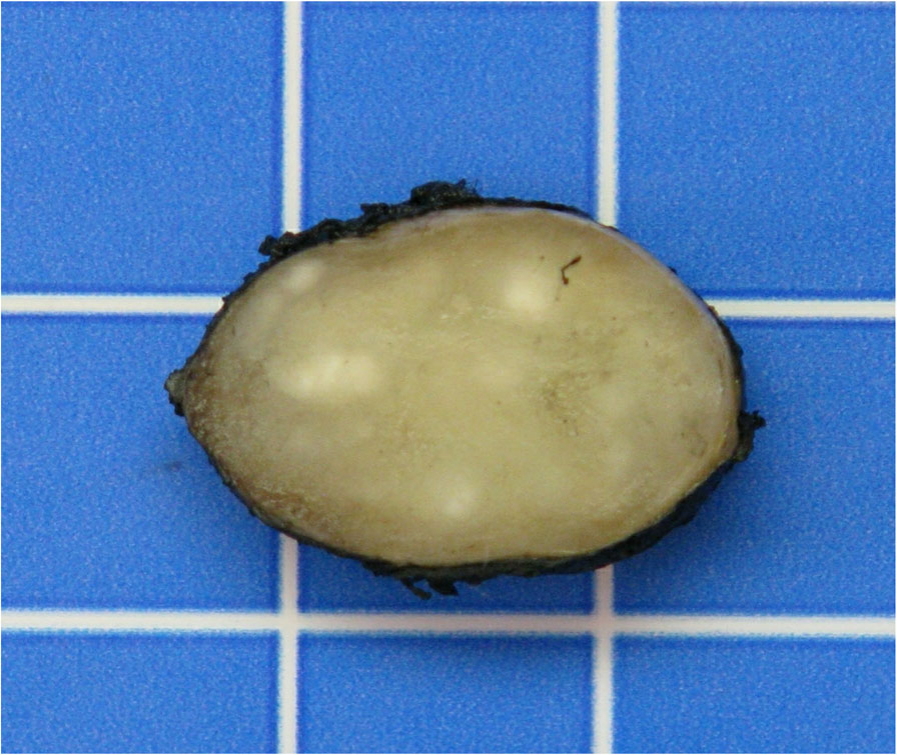
Intra-operative image of the vulvar defect at 6 months after the first surgery. After first surgery, the patient complained of superficial dyspareunia. At clinical examination, a 7 mm skin defect was discovered. Initially conservative management was undertaken with unsatisfying evolution. A surgical correction was proposed and accepted by the patient

A transvaginal ultrasound was performed for pregnancy datation and localization, which confirmed an intrauterine pregnancy of 5 + 3/7 GW. According to the patient’s request, surgical excision of the cyst combined with dilation and curettage was performed. Dilation and curettage were uneventful. During the enucleation of the cyst, we saw a clear mucinous fluid dripping off because the cyst wall was very thin. We applied a Foley catheter swollen with 5 cc of NaCl to maintain a proper cleavage plan of the lesion. Cystectomy was uneventful. The specimen was sent for pathological analysis which identified an 8 × 2 × 1.5-cm cyst of the Bartholin’s gland. Surprisingly, after the excision of the first cyst, we noticed a second smaller mass of 1.5 cm adherent to the right side of the body of the clitoris. The mass had an adipose aspect at sight and consistence at palpation (Fig. 2). We proceeded to the ablation of the second cyst (Fig. 3), respecting the clitoris’ neurovascular bundle. Pathological analysis revealed a 2 × 1.5 × 1.5-cm benign leiomyoma arising from the right side of the clitoris (Fig. 4). The immediate postoperative period was uneventful and the patient was discharged on day 1. Our patient presented with a defect of the cystectomy scar that required surgical correction 6 months after the first surgery, with full recovery (Fig. 5). Considering the genetic implications of an extrauterine leiomyoma, we offered our patient a genetic consultation, which she declined.

## Discussion and conclusions

Uterine leiomyomatas affect 25–30% of women older than 35 [1]. Extrauterine localization is uncommon, with about 120 cases described in the English medical literature worldwide. From a histological point of view, extrauterine leiomyomas’ origin is thought to be from erectile tissue or from the muscular layer of blood vessels [2]. An association between extrauterine leiomyomata and Alport’s syndrome has been described. Alport’s syndrome is a progressive inherited glomerular disease that is associated with renal injury, hearing loss and ocular abnormalities. The etiology is a genetic defect of type IV collagen, which is one of the main components of the glomerular basal membrane. A deletion on Xq22.3 chromosome could enhance both collagen IV deposition and alteration [3]. The current hypothesis is that a deletion involving both COL4A5 and the first two introns of COL4A6 genes could prompt an abnormal deposition of collagen-IV leading to Alport’s syndrome and a dis-regulation of smooth muscle growth leading to leiomyoma formation [4].

As collagen type IV is also an important component of the basement membrane in the esophagus, the same mutation has been reported in familial cases of Alport’s syndrome with esophageal leiomyomatosis. We therefore asked for a gastroenterology consultation, which concluded that, as long as our patient stayed asymptomatic for dysphagia and weight loss, no more investigations were needed.

Recommended imaging studies for leiomyomata are ultrasound, MRI and eventually PET-CT scan in cases of difficult differential diagnosis with sarcomas. The imaging key to diagnosis at MRI is a low T2-weighted signal intensity, similar to that of smooth muscles [1].

The treatment of choice of symptomatic leiomyomata is surgical excision because it allows for pathological examination. Leiomyomata presents a classical microscopic aspect of spindle-bundled cells: immunohistochemistry for smooth muscle markers, such as desmin and smooth muscle actin are positives. Moreover, hormone-induced growth can be demonstrated by targeting estrogen and progesterone receptor. According to Nielsen et al., malignancy criteria are a size of more than 5 cm, more than 1 mitosis per 10 high power fields, nuclear atypia, necrosis and margin infiltration [5]. Vulvar and vaginal masses trigger various differential diagnoses such as cancer, primary or secondary localization of a metastatic spread from vaginal sarcoma [6], Bartholin’s cyst, Müllerian cyst, fibromas, lymphangioma, soft-tissue sarcomas and neurogenic tumors. Epstein Barr virus caused vulvar smooth muscle proliferation in a set of immunocompromised patients [7]. Treatment options are surgical excision with or without GnRH adjuvant therapy. Sun et al. showed in a case series of 21 patients that the recurrence risk of vulvar leiomyomata after surgery is generally low with only one case with two recurrences at 6 months and 5 years post excision [8]. In our case, we did not perform any imaging because clinical examination showed a large but typical vulvar cyst. The clitoral leiomyoma was accidentally found during surgery. Our patient refused any further exam. However, further investigations could have been a thorax MRI and a genetic consultation to rule out other rare localizations of leiomyomata and determine whether she is a carrier of Alport’s syndrome.

This is an original case report, whose strength point is to outline the management of an extrauterine leiomyoma, to raise awareness about the possible association with the Alport’s syndrome or esophageal leiomyomatosis. The limitation of this paper is that, in our case, we were not able to explore these associations as the patient refused any genetic testing.

Nevertheless, in the event of an extrauterine leiomyoma a genetic testing should be proposed and discussed with the patient.

With this article we want to improve recognition of extra uterine leiomyoma by the care givers confronting a fibromuscular para-clitoridean mass. Differential diagnosis in this case should include a para - clitoridean leiomyoma, in addition to other benign or malignant tumors. Should an extrauterine leiomyoma be diagnosed, we advise to extend the familial history in order to detect other women affected.

## Data Availability

The datasets used and/or analysed during the current study available from the corresponding author on reasonable request.

## Abbreviations

GW: Gestational Week
MRI: Magnetic Resonance Imaging
PET-CT: Positron emission tomography–computed tomography
GnRH: Gonadotropin Releasing Hormone
