# Correction to: Clitoral leiomyoma in a premenopausal woman: a case report

**DOI:** 10.1186/s12905-020-00975-x

**Published:** 2020-05-20

**Authors:** Gianmarco Taraschi, Diego Aguiar, Jean Christophe Tille, Patrick Petignat, Jasmine Abdulcadir

**Affiliations:** 1 Department of Obstetrics and Gynaecology, Geneva University Hospitals, 30 Bld de la Cluse, 1211 Geneva, Switzerland; 2 Division of Clinical Pathology, Geneva University Hospital, 1 rue Michel-Servet, 1205 Geneva, Switzerland

**Correction to: BMC Women's Health (2020) 20:89**

**Fig. 1:**
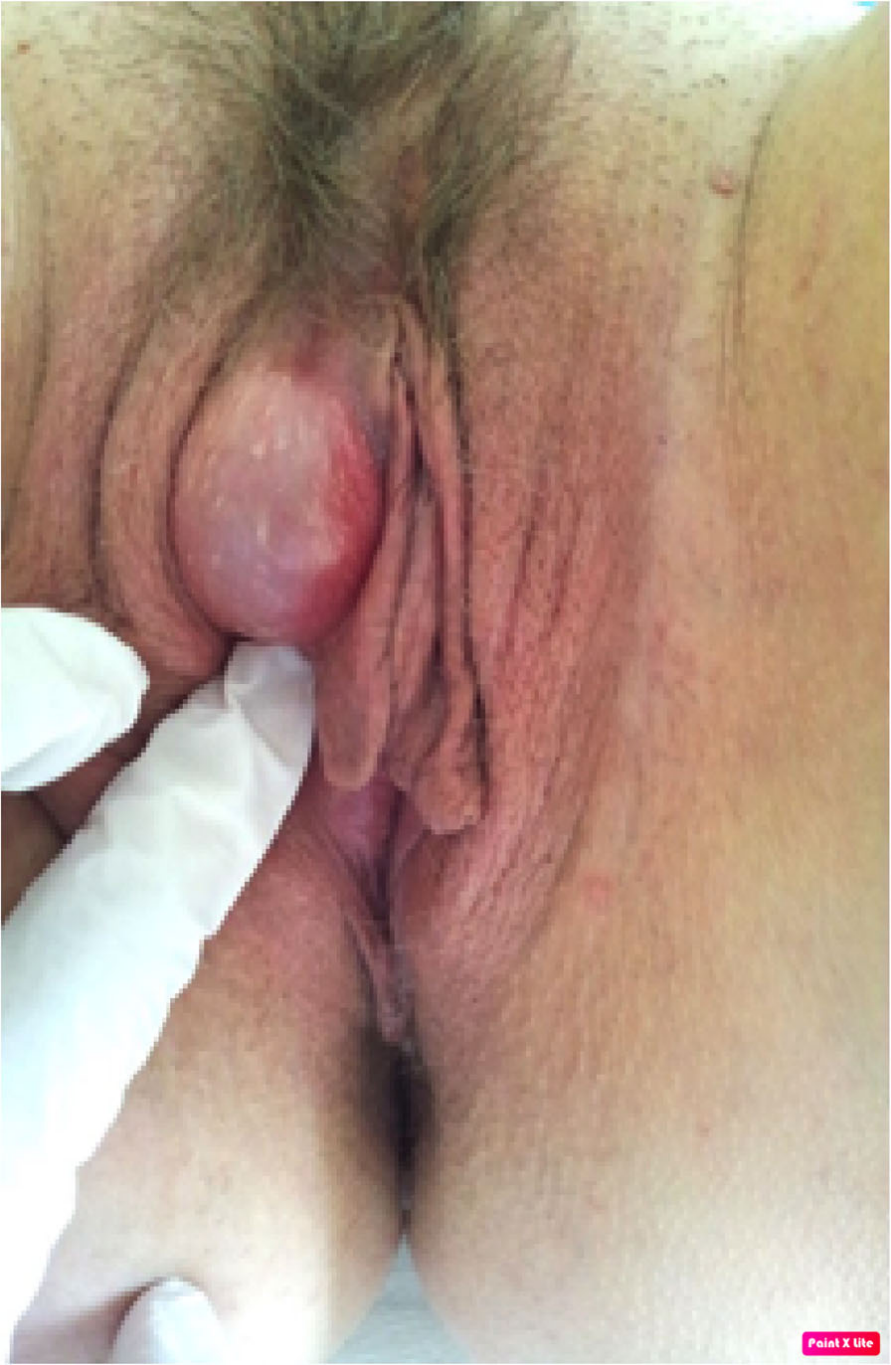
Physical examination, soft bi-lobated fluid-filled swelling of the right interlabial fossa slightly diverting the clitoris on the opposite side. The pathologic examination showed a Bartholin’s gland cyst, which was surprising considering the anatomical localization of this lesion

**Fig. 2:**
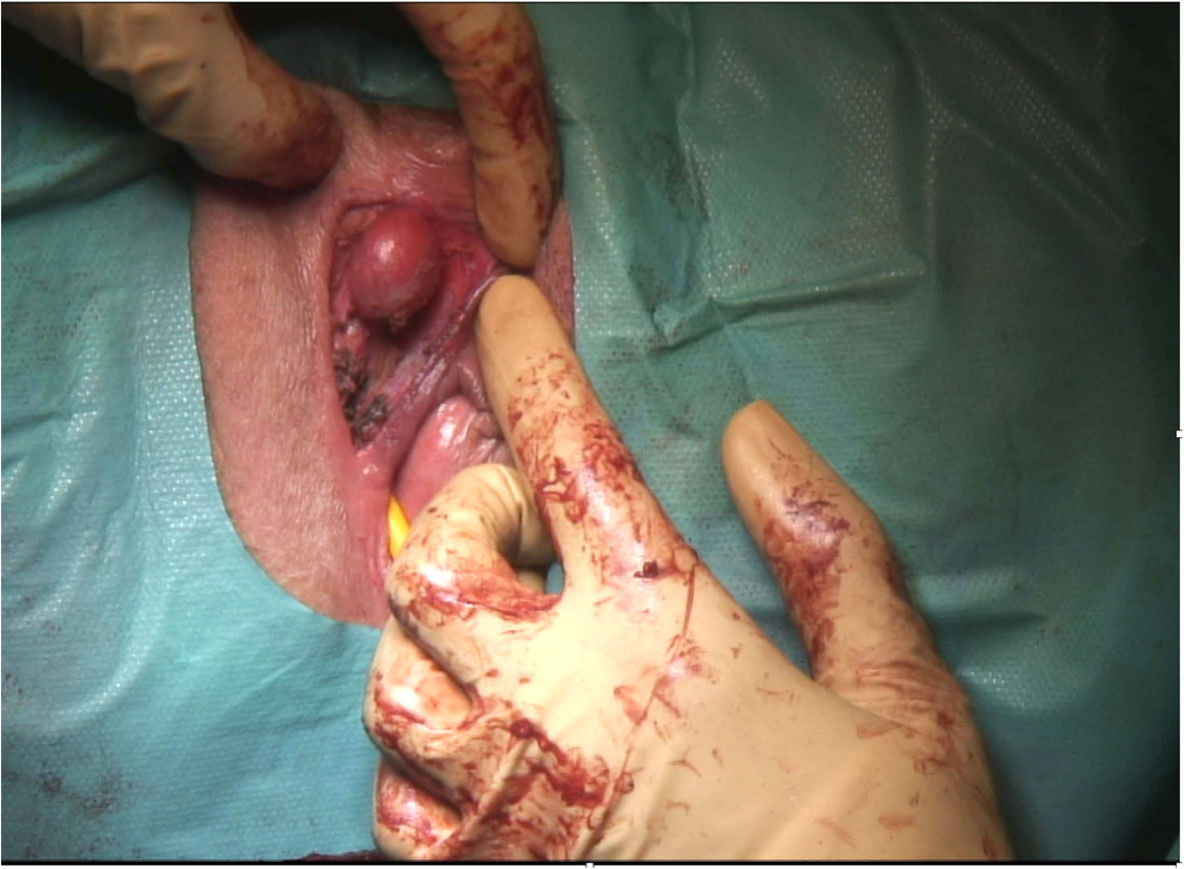
Intra-operative image of the leiomyoma arising from the right side of clitoris after excision of the first Bartholin’s gland cyst

**Fig. 3:**
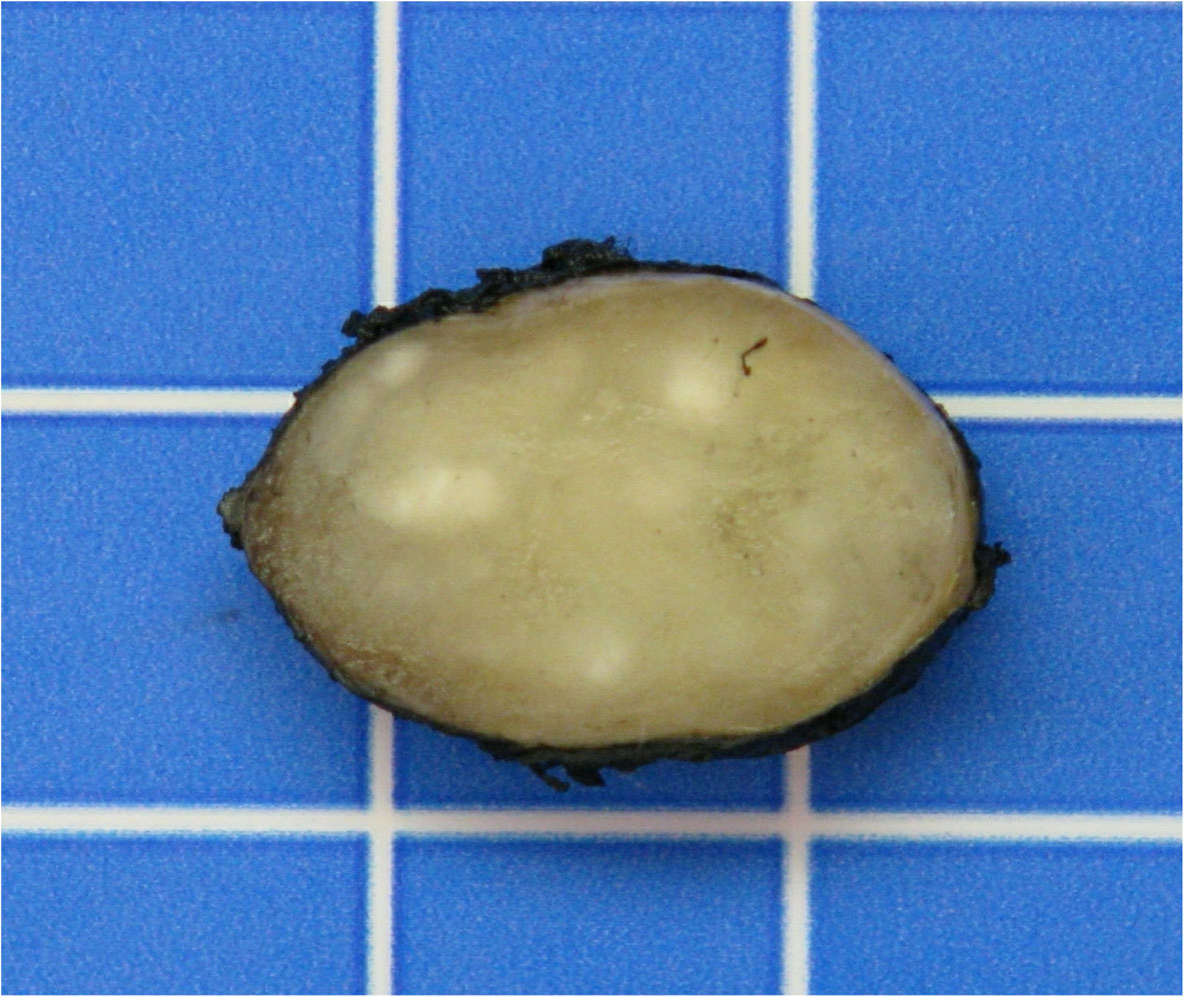
Macroscopic view of the clitoridal mass. (1 square is 1 cm)

**Fig. 4:**
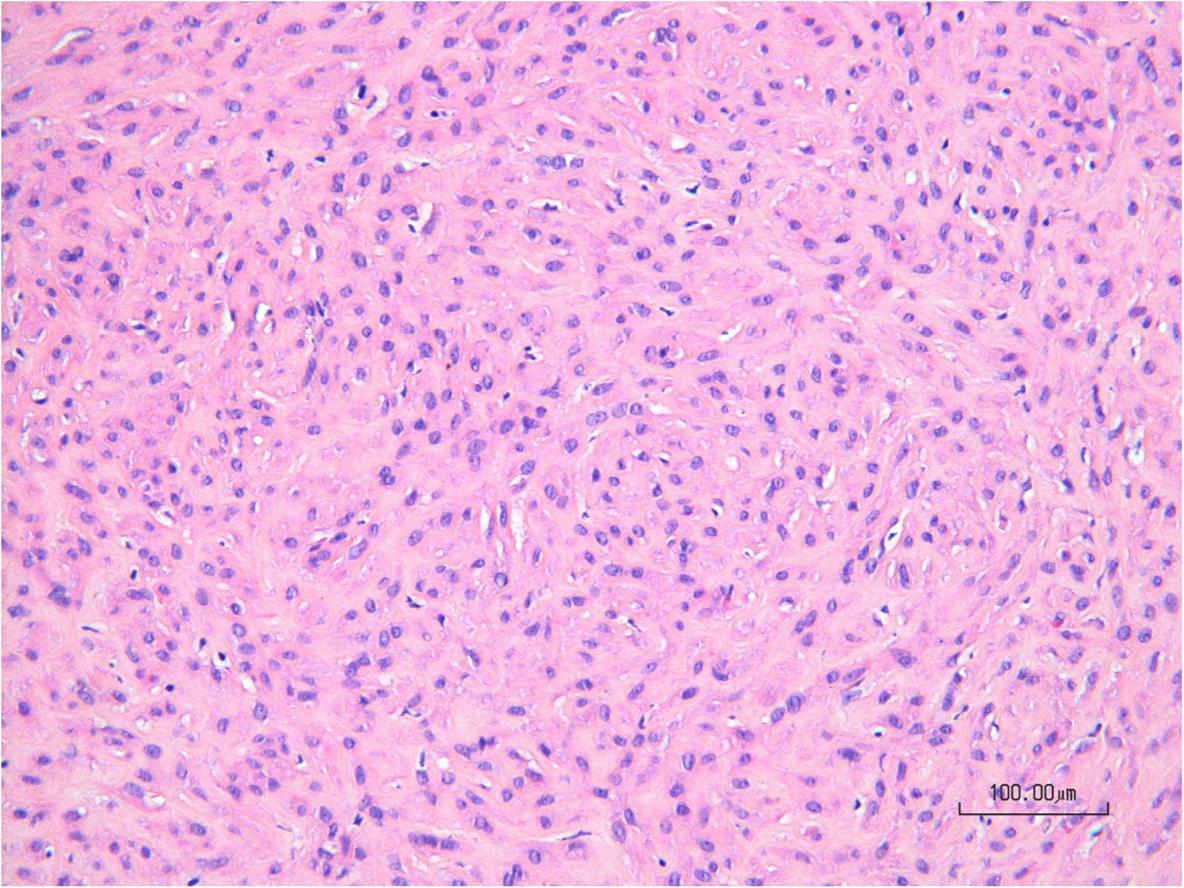
Microscopic aspect of the clitoridal mass showing intersecting fascicles of spindled cells intermixed with collagen (HE, 200x)

**Fig. 5:**
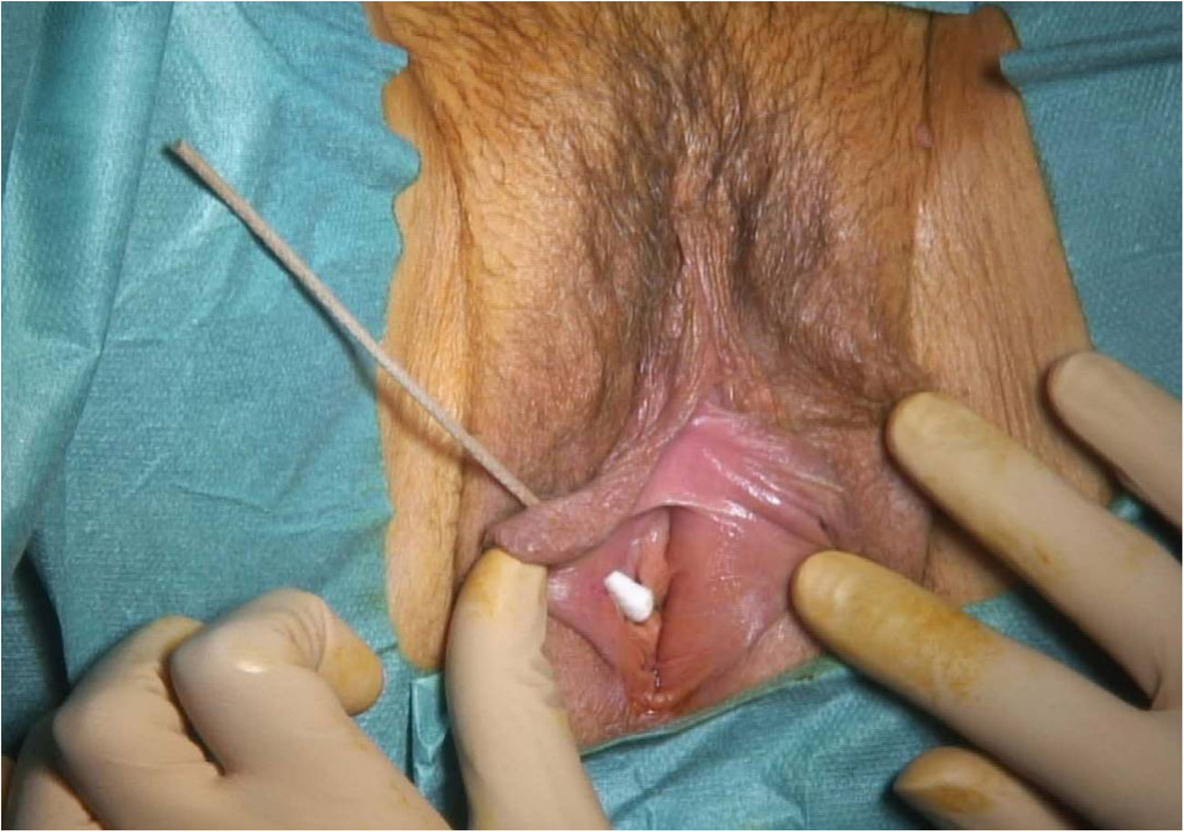
Intra-operative image of the vulvar defect at 6 months after the first surgery. After first surgery, the patient complained of superficial dyspareunia. At clinical examination, a 7mm skin defect was discovered. Initially conservative management was undertaken with unsatisfying evolution. A surgical correction was proposed and accepted by the patient

**https://doi.org/10.1186/s12905-020-00959-x**

Following publication of the original article [1], the authors identified an error in figure order display and figure legends.

The correct figure order and respective legends is shown below:
